# Community-level prevalence and determinants of *Schistosoma mansoni* infection in river-crossing Kebeles of Mizan-Aman Town, Southwest Ethiopia, where mass drug administration targets only school-aged children

**DOI:** 10.1371/journal.pntd.0014506

**Published:** 2026-07-14

**Authors:** Gashaw Temesgen, Zeleke Mekonnen, Mitiku Abera, Delenasaw Yewhalaw

**Affiliations:** 1 Department of Medical Laboratory Sciences, Mizan Aman College of Health Sciences, Mizan-Aman, Ethiopia; 2 School of Medical Laboratory Sciences, Institute of Health, Jimma University, Jimma, Ethiopia; 3 Department of Medical Laboratory Sciences, College of Medicine and Health Sciences, Mizan-Tepi University, Mizan-Aman, Ethiopia; 4 Tropical and Infectious Diseases Research Centre, Institute of Health, Jimma University, Jimma, Ethiopia; Washington University School of Medicine, UNITED STATES OF AMERICA

## Abstract

**Introduction:**

Schistosomiasis remains a major neglected tropical disease, particularly in sub-Saharan Africa, where *Schistosoma mansoni* (*S. mansoni*) causes intestinal schistosomiasis through contact with cercariae-infested freshwater. In Ethiopia, control relies mainly on school-based mass drug administration (MDA) targeting school-aged children (SAC), while adults and other at-risk groups are often untreated. This study assessed the community-level prevalence and determinants of *S. mansoni* infection in Mizan-Aman Town following implementation of SAC-focused MDA.

**Methods:**

A community-based cross-sectional study was conducted from October 2021 to February 2022 in Mizan-Aman Town. A total of 349 participants from selected households were enrolled using systematic sampling. Socio-demographic and risk factor data were collected through interviewer-administered questionnaires. Stool samples (~5 g) were collected and examined using duplicate Kato–Katz thick smears (41.7 mg template). Bivariate and multivariable logistic regression analyses were performed to identify associated factors, with significance set at p < 0.05.

**Results:**

The prevalence of *S. mansoni* infection was 23.2% (95% CI: 18.9–28.0). Poly parasitism was observed in 12.3% of participants and the overall intestinal parasitic infection prevalence was 50.7%. Moreover, use of river water for domestic purposes (AOR = 5.86, 95% CI: 2.90–11.83), bathing in rivers (AOR = 2.98, 95% CI: 1.32–6.73), swimming in rivers (AOR = 2.62, 95% CI: 1.28–5.34), and farmland proximity to rivers (AOR = 2.28, 95% CI: 1.10–4.75) were statistically significant predictors. Age and sex were not significantly associated with infection.

**Conclusion:**

The current study revealed a moderate prevalence of *S. mansoni*, suggesting that untreated community members may serve as reservoirs of infection for SAC and indicating ongoing transmission despite school-based MDA. Therefore, expanding treatment to include adults, together with improved water, sanitation, and hygiene interventions and continuous epidemiological surveillance, is essential for sustainable reduction of transmission.

## Author summary

Schistosomiasis is a parasitic disease that affects many people in sub-Saharan Africa, including Ethiopia. It is caused by blood flukes (schistosomes), particularly *S. mansoni* in many endemic areas, especially in areas where people frequently come into contact with schistosome infested river and lack adequate sanitation and hygiene. In Ethiopia including Mizan-Aman Town, control efforts mainly focus on school-based mass drug administration targeting school-aged children at school only, while other community members are often left untreated. Despite these efforts, the disease may continue to circulate within the wider community.

In the present study, we examined stool samples using the specialized laboratory techniques (Kato-Katz technique) from 349 community members, revealing that 23.2% were infected with *S. mansoni,* that means nearly one in four people in the community was infected. We also assessed behaviors and environmental factors that increase the risk of infection. We identified activities such as using river water for household purposes, bathing, and swimming in rivers, as well as living near farmland close to rivers, increased the risk of infection.

Overall, these findings show that focusing treatment only on school-aged children is not sufficient to interrupt transmission. Untreated adults and other community members may continue to act as sources of infection. Therefore, expanding treatment to include the whole community, together with improving access to clean water, sanitation, and hygiene, is essential. At the same time, strengthening public health education and regular monitoring can help reduce infection and improve overall community health.

## Introduction

Schistosomiasis is one of the most prevalent neglected tropical diseases (NTDs) worldwide, posing a significant public health burden in many low and middle-income countries, mainly in sub-Saharan Africa [1,2]. The disease is caused by blood flukes of the genus Schistosoma, with *Schistosoma mansoni* being the predominant species responsible for intestinal schistosomiasis in Africa [3]. Transmission occurs through skin contact with freshwater contaminated by cercariae released from infected Biomphalaria snail intermediate hosts, making the disease closely associated to environmental, behavioral, and socio-economic factors [4,5].

Globally, around 230 million people are estimated to be infected with schistosomes and 700 million people living in areas at risk of infection. More than 90% of the global schistosomiasis burden is in Sub-Saharan Africa countries [2,6]. Ethiopia is one of the countries where *S. mansoni* infection remains a major public health problem, particularly in areas where people use rivers, streams, irrigation schemes, and dams which facilitates snail breeding and human water contact [7,8]. As reported in different National and regional studies, there are varying prevalence levels, reflecting ecological diversity and differences in local transmission dynamics [9]. SAC and young adults are often affected more, than others. This is because of increased exposure during swimming, bathing, washing clothes, and agricultural activities [10,11]. However, evidence suggests that populations other than SAC, particularly individuals engaged in river-related activities such as irrigation, fishing, and washing, are also at increased risk of schistosomiasis infection [1].

Chronic *S. mansoni* infection may result in significant morbidity, such as abdominal pain, diarrhea, hepatosplenomegaly, periportal fibrosis, anemia, malnutrition, growth retardation, and impaired cognitive development [4,12]. All these complications can affect educational achievement, work productivity, and community development, indicating the importance of effective control measures against *S mansoni* infection [13,14].

Mass drug administration with praziquantel targeting primarily to reduces morbidity by killing adult worms and lowering egg output in human hosts is the cornerstone of schistosomiasis control. It is assumed that reducing egg excretion may also partially reduce environmental contamination and transmission, however, the effect is usually incomplete in some highly endemic areas for *S. mansoni* infection particularly, where adults and other at-risk groups are often untreated [1,15]. In some areas reinfection occurs most of the time, and transmission persist, especially, where human-water contact persists and snail intermediate hosts remain abundant [16]. Consequently, the community level epidemiological profile of *S. mansoni* infection including prevalence, and population groups at risk may remain unchanged or change only slowly over time even in areas under MDA programs.

Recently, the World Health Organization (WHO) NTD Roadmap 2021–2030 has shifted the global strategic focus for schistosomiasis from morbidity control toward the interruption of transmission and eventual elimination as a public health problem in endemic countries [17]. Achieving these targets requires robust community-level epidemiological evidence to identify residual transmission foci, understand infection patterns among different population groups, and evaluate whether current control strategies are sufficient to interrupt transmission. In settings where preventive chemotherapy is primarily targeted to SAC, monitoring infection among the wider community is particularly important for identifying potential reservoirs that may sustain ongoing transmission [1,17].

Despite a number of earlier studies conducted in Ethiopia, regular and current community level epidemiological data are crucial, particularly in regions where MDA programs are still in place. These kinds of data are essential for tracking the effectiveness of programs, locating hotspots for residual transmission, assessing the results of interventions, and directing complementary strategies such as expanding MDA to other at-risk groups, snail control and improvements in water, sanitation, and hygiene (WASH) [18,19]. The findings of the present study therefore provide important evidence to support adaptive, evidence-based decision-making and sustain progress toward the control and eventual elimination of schistosomiasis.

In Southwest Ethiopia, particularly in Mizan-Aman and the Bench-Sheko Zone, local community level epidemiological data on *S. mansoni* infection with regard to MDA program remains limited. The presence of freshwater bodies, water contact activities, and environmental factors in the area point to the possibility of continuous transmission, which supports the necessity for an updated epidemiological assessment after MDA program in the community. Thus, the purpose of this study was to determine the community-level prevalence and determinants of *S. mansoni* infection in a setting where MDA targets only SAC in river-crossing Kebeles of mizan Aman, Bench-Sheko Zone, Southwest Ethiopia. It is anticipated that the results would be valuable baseline information for local public health authorities and contribute to improved, evidence-based control strategies.

## Materials and methods

### Ethics statement

The study was conducted following Ethical approval obtained from the Institutional Review Board of the Institute Health of Jimma University (Ref. No. Rf/IHRPGn/187). Permission was secured from local health authorities. Written informed consent was obtained from adult participants and guardians of minors; assent was obtained from children aged 12–17 years. Participants found positive for *S. mansoni* or other helminths were treated with praziquantel and albendazole according to national guidelines.

### Study area and period

The study was carried out in Mizan-Aman Town, Bench-Sheko Zone, Southwest Ethiopia, from October 2021 to February 2022. The town is located approximately 561 km southwest of Addis Ababa. capital city of Ethiopia and lies at an altitude of 1,451–1,753 m above sea level. It has a humid tropical climate, with annual rainfall ranging from 400 to 2,000 mm and average temperatures of 15.1°C to 27°C, conditions that favor the survival of freshwater snail intermediate hosts, particularly Biomphalaria species, which are essential for *S. mansoni* transmission. Mizan-Aman is crossed by several perennial and seasonal freshwater bodies, including rivers, streams, and swampy areas, widely used for bathing, washing, water collection, irrigation, and recreation. This frequent human–water contact creates favorable conditions for the continued transmission of intestinal schistosomiasis.

Mizan-Aman Town has a population of 123,005 (63,963 males and 59,042 females) distributed across five Kebeles. The population engages mainly in small-scale farming, daily labor, petty trade, and public service employment. In peri-urban areas, inadequate sanitation, limited access to safe water, and open defecation persist, increasing the risk of *S. mansoni* transmission. Kometa and Shasheka Kebeles were selected for this study because they are crossed by rivers suspected as transmission sites, namely the Shonga and Agu Rivers, respectively. Kometa has 7,903 residents and 1,665 households, while Shasheka has 11,118 residents and 2,334 households.

### Study design and population

A community-based cross-sectional study was conducted among individuals of all age groups who had lived in the study area for at least six months. Participants who provided informed consent and had not received anti-helminthic treatment within the past two months were included in the study.

### Sample size determination and sampling techniques

The sample size was determined using a single population proportion formula, $n=\frac{Z^{2}*p\left(1-p\right)}{d^{2}}$ assuming a prevalence of *S. mansoni* infection of 9.3% based on a previous community-based study in Southwest Ethiopia [20], a 95% confidence level (Z = 1.96), and a 3% margin of error. The initial sample size was 360. Since the total number of households in the selected kebeles was less than 10,000, a finite population correction was applied, resulting in a sample size of 331. After adding a 10% non-response rate, the final sample size was 364.

Kometa and Shasheka Kebeles were selected purposely due to their proximity to rivers suspected to be sources of infection and high human water contact. Subsequently, a systematic sampling technique was employed to select households from the selected Kebeles, with proportional allocation applied to determine the number of households sampled from each Kebele. Based on this approach, 152 households from Kometa and 212 households from Shasheka were included in the study. The first household was selected using a lottery method, and subsequent households were selected at a regular sampling interval (k) of 11 until the required sample size was reached. The sampling interval was calculated as k = N/n = 3,999/364 ≈ 11. From each selected household, one eligible individual was chosen randomly.

### Data collection

#### Socio-demographic and risk factors.

A semi-structured questionnaire was initially prepared in English, translated into Amharic, and then back-translated into English to ensure consistency and accuracy. The questionnaire was pretested before the actual data collection, and necessary modifications were made accordingly. The questionnaire included items addressing the socio-demographic characteristics of the participants and potential risk factors associated with *S. mansoni* infection. Data were collected through interviewer-administered questionnaires after obtaining written informed consent from each participant.

### Parasitological examination

Approximately 5 g (thumb-sized) of stool specimen was collected from each participant using a clean, dry, wide-mouthed, and leak-proof container. All collected samples were transported to the Mizan Aman public health regional laboratory using a cold box and processed using the two-slide Kato–Katz technique (41.7 mg template) [21]. After preparing duplicate thick smears following the WHO (2019) standard operating procedures, the slides were left at room temperature for at least one hour to allow clearing of fecal material prior to examination, and all slides were examined within 24 hours of preparation. However, for hookworm eggs, the smears were examined within 30 minutes of preparation to avoid egg clearing. Each specimen was independently examined by two senior laboratory technologists. In cases of discrepancy between the two readings, the slides were re-examined by a third experienced laboratory technologist, and the final result was recorded based on consensus.

To determine the egg per gram of stool in each Kato-Katz slide, the total counted parasite per slide was multiplied by 24 for each slide (24*41.7 mg template = 1000mg(1g)). The intensity of *S. mansoni* infection was determined according to WHO intensity classes (light, moderate and heavy intensity) [22].

### Data management and analysis

Data were entered into EpiData version 4.6.06 and exported to SPSS version 26.0 for statistical analysis after checking for completeness and consistency. Descriptive statistics, including frequencies, proportions, and mean, were used to summarize the data. Bivariate logistic regression analysis was performed to assess the association between each independent variable and the outcome variable. Variables with a p-value < 0.25 in the bivariate analysis were entered into the multivariable logistic regression model to control for potential confounders. Adjusted odds ratios (AORs) with 95% confidence intervals (CIs) were computed to determine the strength of associations. Variables with a p-value < 0.05 in the multivariable logistic regression analysis were considered statistically significant determinants of the outcome variable.

## Results

### Socio-demographic characteristics of study participants

In the present study a total of 349 participants were enrolled in the study, yielding a response rate of 96%. Males accounted for 52.1%, and females for 47.9% of the study participants, with a median age of 20 years. Nearly all participants (97.4%) had lived in the study area for at least one year. Almost half were students, and Protestant Christianity was the predominant religion (Table 1).

**Table 1:** Socio-demographic characteristics of study participants in Mizan Aman Town, Bench Sheko Zone, Southwest Ethiopia (n = 349).

Variable	Category	Frequency (n)	Percentage (%)
**Sex**	Male	182	52.1
	Female	167	47.9
**Age (years)**	1–4	36	10.3
	5–9	43	12.3
	10–14	49	14.0
	15–19	46	13.2
	20–24	54	15.5
	≥25	121	34.7
**Length of stay**	>6 months	9	2.6
	≥1 year	340	97.4
**Education**	Preschool	48	13.8
	No formal education	39	11.2
	Primary school	166	47.6
	Secondary & above	96	27.5
**Religion**	Orthodox	110	31.5
	Muslim	92	26.4
	Protestant	147	42.1
**Monthly income (ETB)**	301–500	117	33.5
	501–1000	120	34.4
	>1000	112	32.1
**Occupation**	Unemployed	43	12.3
	Housewife	67	19.2
	Student	167	47.9
	Labourer	12	3.4
	Farmer	32	9.2
	Merchant	14	4.0
	Government employee	14	4.0

### Prevalence of *S. mansoni* and other intestinal parasites

Out of 349 screened study participants the overall prevalence of *S. mansoni* infection was 81 (23.2%); 95% CI: (18.9-28.0). Mono-infection with *S. mansoni* accounted for 10.9%, while 12.3% of participants had *S. mansoni* co-infection with other helminths. Overall, 50.7% of participants were infected with at least one intestinal parasite (Table 2).

**Table 2:** Prevalence of S. mansoni, soil-transmitted helminths, and other intestinal parasite infections among study participants in Mizan Aman, Southwest Ethiopia (n = 349).

Infection category	Parasite species	Frequency (n)	Percentage (%)
***S. mansoni***	Mono-infection	38	10.9
	Co-infection	43	12.3
	**Total**	**81**	**23.2**
**STH mono-infection**	*Ascaris lumbricoides*	15	4.3
	*Trichuris trichiura*	49	14.0
	Hookworm	7	2.0
	**Total**	**71**	**20.3**
**Other parasites**	*Taenia* spp.	3	0.9
	*Hymenolepis nana*	2	0.6
	**Total**	**5**	**1.4**
**Double infections**	*A. lumbricoides* & *T. trichiura*	13	3.7
	*A. lumbricoides* & *S. mansoni*	1	0.3
	Hookworm & *T. trichiura*	6	1.7
	*S. mansoni* & *T. trichiura*	36	10.3
	Hookworm & *S. mansoni*	1	0.3
	**Total**	**57**	**16.3**
**Multiple infections**	Three or more species	6	1.7

### Infection Intensity of *S. mansoni* Infection Among Study Participants

Among the 349 study participants examined, 81 (23.2%) were infected with *S. mansoni*. Regarding infection intensity, 64 (18.3%) participants had light-intensity infection, 13 (3.7%) had moderate-intensity infection, and 4 (1.2%) had heavy-intensity infection. Light-intensity infection was the predominant category among the study participants (Table 3).

**Table 3:** Prevalence of S. mansoni infection intensity among study participants in Mizan Aman, Southwest Ethiopia (n = 349).

Parasite species	Number of infected *participants* n (%)	Intensity of infection		
		Light n (%)	Moderate n (%)	Heavy n (%)
*S. mansoni*	81(23.2)	64 (18.3)	13 (3.7)	4(1.2)

### Factors associated with *S mansoni* infection

In this study variables with p < 0.25 in bivariate analysis were included in the multivariable logistic regression model (Table 4a). In the final model, use of river water, bathing in rivers, swimming habits, and farmland proximity to rivers remained statistically associated with *S. mansoni* infection. Participants who used river water for domestic purposes were nearly six times more likely to be infected compared to those were not used. Individuals who reported bathing in rivers had approximately three times higher odds of infection. Similarly, participants with a habit of swimming in rivers were more than two and a half times more likely to be infected. Furthermore, those who had farmland located near rivers were 2.28 times more likely to be infected compared to those without such exposure. Other factors, including sex, age, and river crossing, did not show statistically significant associations with *S. mansoni* infection in the multivariable analysis (Table 4b).

**Table 4:** (a) Bivariate logistic regression analysis of factors associated with S. mansoni infection in Mizan Aman, Southwest Ethiopia (n = 349). (b) Multivariable logistic regression analysis of factors associated with S. mansoni infection in Mizan Aman, Southwest Ethiopia (n = 349).

(a) Bivariate logistic regression analysis of factors associated with S. mansoni infection in Mizan Aman, Southwest Ethiopia (n = 349).					
Variable	Category	*S. mansoni* +ve n (%)	*S. mansoni* -ve n (%)	COR (95% CI)	p-value
**Sex**	Male	48 (26.4)	134 (73.6)	**1.455** (0.879–2.407)	0.145*
	Female	33 (19.8)	134 (80.2)	1	
**Age (years)**	1–4	5 (13.9)	31 (86.1)	1	
	5–9	19 (44.2)	24 (55.8)	**4.908** (1.601–15.044)	0.005*
	10–14	15 (30.6)	34 (69.4)	**2.735** (0.890–8.409)	0.079*
	15–19	15 (32.6)	31 (67.4)	**3.000** (0.971–9.268)	0.056*
	20–24	11 (20.4)	43 (79.6)	**1.586** (0.500–5.027)	0.433
	≥25	16 (13.2)	105 (86.8)	**0.945** (0.320–2.785)	0.918
**Water source for domestic use**	Pipe	4 (20.0)	16 (80.0)	**1.937** (0.599–6.263)	0.270
	Spring	19 (26.0)	54 (74.0)	**1.660** (0.520–5.299)	0.392
	River	42 (53.8)	36 (46.2)	**9.851** (5.400–17.969)	<0.001*
	Well	16 (9.0)	162 (91.0)	1	
**Bathing in river**	Yes	69 (35.9)	123 (64.0)	**6.778** (3.509–13.094)	<0.001*
	No	12 (7.6)	145 (92.4)	1	
**Swimming**	Yes	34 (40.5)	50 (59.5)	**4.934** (2.893–8.413)	<0.001*
	No	47 (17.7)	218 (82.3)	1	
**Crossing river**	Yes	42 (38.5)	67 (61.5)	**3.231** (1.928–5.413)	<0.001*
	No	39 (16.3)	201 (83.8)	1	
**Farmland near river**	Yes	38 (42.7)	51 (57.3)	**3.760** (2.208–6.404)	<0.001*
	No	43 (16.5)	217 (83.5)	1	
**Table 4b. Multivariable logistic regression analysis of factors associated with S. mansoni infection in Mizan Aman, Southwest Ethiopia (n = 349).**					
**Variable**	**Category**	***S. mansoni* +ve n (%)**	***S. mansoni* -ve n (%)**	**AOR (95% CI)**	**p-value**
**Sex**	Male	48 (26.4)	134 (73.6)	**0.810** (0.418–1.571)	**0.533**
	Female	33 (19.8)	134 (80.2)	1	**—**
**Age (years)**	1–4	5 (13.9)	31 (86.1)	1	**—**
	5–9	19 (44.2)	24 (55.8)	**1.366** (0.356–5.249)	**0.650**
	10–14	15 (30.6)	34 (69.4)	**0.571** (0.145–2.252)	**0.423**
	15–19	15 (32.6)	31 (67.4)	**0.597** (0.151–2.368)	**0.463**
	20–24	11 (20.4)	43 (79.6)	**0.545** (0.138–2.144)	**0.385**
	≥25	16 (13.2)	105 (86.8)	**0.302** (0.082–1.118)	**0.073**
**Water source for domestic use**	Pipe	4 (20.0)	16 (80.0)	**2.562** (0.672–9.763)	**0.168**
	Spring	19 (26.0)	54 (74.0)	**0.997** (0.274–3.634)	**0.997**
	River	42 (53.8)	36 (46.2)	**5.853** (2.897–11.826)	<0.001**
	Well	16 (9.0)	162 (91.0)	1	**—**
**Bathing in river**	Yes	69 (35.9)	123 (64.0)	**2.979** (1.318–6.730)	0.009**
	No	12 (7.6)	145 (92.4)	1	**—**
**Swimming**	Yes	34 (40.5)	50 (59.5)	**2.617** (1.284–5.336)	0.008**
	No	47 (17.7)	218 (82.3)	1	**—**
**Crossing river**	Yes	42 (38.5)	67 (61.5)	**1.418** (0.692–2.908)	**0.340**
	No	39 (16.3)	201 (83.8)	1	**—**
**Farmland near river**	Yes	38 (42.7)	51 (57.3)	**2.280** (1.095–4.745)	0.028**
	No	43 (16.5)	217 (83.5)	1	**—**

* Indicates the significant variables p < 0.25, COR = crude odds ratio, *+ ve* = *positive, -ve = Negative***,** 1 = reference group

** Indicates the significant variables P < 0.05, AOR = adjusted odds ratio, *+ ve* = *positive, -ve = Negative***,** 1 = reference group

## Discussion

Schistosomiasis is one of the NTD transmitted by a freshwater snail host, that remains a public health problem in many parts of sub-Saharan Africa countries, including Ethiopia [23,24]. Schistosomiasis control strategies in Ethiopia primarily rely on periodic MDA with praziquantel, which is mainly delivered through school-based platforms and targets SAC as the primary risk group for morbidity [24]. Although this approach has been effective in reducing infection intensity and morbidity among treated children, it does not fully interrupt transmission when other exposed population groups such as preschool children, out-of-school adolescents, and adults at risk remain untreated and continue to contaminate freshwater bodies with parasite eggs [1,25]. As a result, persistent community-level transmission has been reported in several endemic settings despite repeated rounds of school-based MDA [19,26,27]. In this context, the present community-based study was conducted to generate up-to-date epidemiological evidence on the prevalence and determinants of *S. mansoni* infection in Mizan-Aman Town.

This community-based study revealed that *S. mansoni* infection remains a moderate public health problem in Mizan-Aman Town community, with an overall prevalence of 23.2%. As per WHO classification, areas with a prevalence range between 10% and 50% are considered moderately endemic, showing sustained transmission in the community despite ongoing control efforts [25]. In Ethiopia, schistosomiasis control depends majorly on school-based MDA focusing only SAC, whereas adults and other community members are not routinely included, with the exception of small, focused campaigns [24]. Although this strategy can reduce morbidity among SAC, its limited coverage of other at-risk populations may reduce its effectiveness in interrupting transmission and achieving elimination at the community level, as suggested by the findings of the present study.

Moreover, the moderate prevalence of *S. mansoni* observed in the general community suggests that transmission may still be ongoing in the study area despite the implementation of MDA targeting SAC. This may be due to continued human-water contact, the presence of suitable snail intermediate hosts, and the exclusion of other at-risk groups, including preschool children, out-of-school adolescents, and adults, from routine MDA programs. These untreated populations may serve as reservoirs of infection and contribute to continued transmission within the community. Similar observations have been reported from the Abbey and Didessa Valleys of western Ethiopia and the Lake Chamo area in the southern Rift Valley, where persistent *S. mansoni* transmission was documented despite ongoing MDA interventions [19,28,29].

Furthermore, in the current study, the high prevalence of the overall intestinal parasitic infections (50.7%) and the substantial proportion of *S. mansoni* co-infections with soil-transmitted helminths reflect ongoing environmental contamination and overlapping transmission routes. Similar patterns have been reported from several endemic areas of Ethiopian and East African settings, where inadequate sanitation, unsafe water sources, and poor hygiene practices facilitate polyparasitism [20–33]. Moreover, Polyparasitic infections are known to exacerbate morbidity, including anemia, undernutrition, and reduced work productivity, particularly among untreated adult populations [34].

Although achieving interruption of *S. mansoni* transmission still requires substantial effort, the present study found that infection was predominantly of light intensity, affecting 18.3% of the study participants, whereas moderate and heavy-intensity infections were less common, accounting for 3.7% and 1.2% of participants, respectively. The predominance of light-intensity infections may indicate a reduced parasite burden in the community, possibly reflecting the impact of ongoing schistosomiasis control interventions, including MDA, health education, and increased community awareness. However, the proportion of heavy-intensity infections (1.2%) remains slightly above the WHO threshold of <1% heavy-intensity infection required for the elimination of schistosomiasis as a public health problem. Therefore, continued and strengthened control efforts are necessary to further reduce infection intensity and move toward achieving the elimination target. [17].

In this study, factors such as use of river water for domestic purposes, bathing and swimming in rivers, and having farmland located near rivers were significantly associated with *S. mansoni* infection. These findings are supported with the established transmission dynamics of intestinal schistosomiasis, which depends on repeated human contact with cercariae-infested freshwater [4]. Adults are particularly vulnerable due to occupational and domestic activities such as farming, washing, irrigation, and fishing, which involve prolonged water contact [35]. Because these high-risk adult groups are not systematically covered by school-based MDA, they may serve as persistent reservoirs of infection, sustaining transmission within the community [1].

Although praziquantel-based MDA is effective in reducing worm burden and egg output among treated individuals, its impact on transmission interruption is limited when treatment coverage does not include the entire at-risk population [1,17]. Reduced egg excretion following treatment can lower environmental contamination temporarily; however, untreated individuals particularly, out-of-school adolescents and adults continue to discharge parasite eggs into freshwater bodies, maintaining the life cycle of *S. mansoni* and facilitating reinfection of treated children [17,36]. Several field studies have shown that morbidity control can be achieved with school-based MDA, but transmission elimination generally requires community-wide treatment or adding other interventions [37,38].

The lack of significant association between *S. mansoni* infection and age group in the multivariable analysis of present study suggests widespread exposure across demographic groups. Similar findings have been reported from endemic areas where adults remain untreated and experience frequent water contact, resulting in infection patterns that extend beyond SAC [39,40]. This underscores the limitation of relying only on school-based prevalence data to assess transmission intensity and program impact [41].

These findings highlight the critical importance of frequent and up-to-date epidemiological surveillance, particularly in areas implementing school-based MDA. WHO and other experts have emphasized that routine community-level surveys are essential to monitor residual transmission, assess reinfection patterns, and evaluate the long-term effectiveness of control programs [1,17]. Without updated epidemiological data that include adults and other high-risk groups, control programs may underestimate ongoing transmission and overestimate progress toward elimination goals [19].

### Study Limitations

The limitation of this study is its cross-sectional design, which restricts our ability to establish causal relationships between variables. Since data were collected at a single point in time, we cannot determine whether the exposures preceded the outcomes. Future longitudinal studies are needed to confirm these associations and provide a deeper understanding of the causal mechanisms involved.

### Conclusion

In general, this community-based study revealed a moderate prevalence of *S. mansoni* among the general community in Mizan-Aman Town, indicating ongoing transmission at the community level. This finding also underscores that the limitations of relying solely on school-based MDA in settings where adults have substantial exposure and often remain untreated. Although MDA can effectively reduce morbidity among SAC, sustainable interruption of transmission will likely require expanded treatment coverage that includes the adult population, alongside integrated water, sanitation, and hygiene interventions and continuous epidemiological monitoring to inform adaptive control strategies.
